# Comprehensive Analysis of the Cadmium Tolerance of Abscisic Acid-, Stress- and Ripening-Induced Proteins (ASRs) in Maize

**DOI:** 10.3390/ijms20010133

**Published:** 2019-01-01

**Authors:** Jie Zhang, Qiusha Zhu, Haijuan Yu, Liang Li, Guoqiang Zhang, Xi Chen, Mingyi Jiang, Mingpu Tan

**Affiliations:** National Key Laboratory of Crop Genetics and Germplasm Enhancement, College of Life Sciences, Nanjing Agricultural University, Nanjing 210095, China; 2017116037@njau.edu.cn (J.Z.); 2017816148@njau.edu.cn (Q.Z.); 2014116028@njau.edu.cn (H.Y.); 2014116029@njau.edu.cn (L.L.); 2015116027@njau.edu.cn (G.Z.); xi.chen@njau.edu.cn (X.C.); myjiang@njau.edu.cn (M.J.)

**Keywords:** ABA-stress-ripening (ASR), subcellular localization, cadmium stress, maize

## Abstract

In plants, abscisic acid-, stress-, and ripening-induced (ASR) proteins have been shown to impart tolerance to multiple abiotic stresses such as drought and salinity. However, their roles in metal stress tolerance are poorly understood. To screen plant Cd-tolerance genes, the yeast-based gene hunting method which aimed to screen Cd-tolerance colonies from maize leaf cDNA library hosted in yeast was carried out. Here, maize *ZmASR1* was identified to be putative Cd-tolerant through this survival screening strategy. In silico analysis of the functional domain organization, phylogenetic classification and tissue-specific expression patterns revealed that maize *ASR1* to *ASR5* are typical *ASRs* with considerable expression in leaves. Further, four of them were cloned for testifying Cd tolerance using yeast complementation assay. The results indicated that they all confer Cd tolerance in Cd-sensitive yeast. Then they were transiently expressed in tobacco leaves for subcellular localization analysis and for Cd-challenged lesion assay, continuously. The results demonstrated that all 4 maize ASRs tested are localized to the cell nucleus and cytoplasm in tobacco leaves. Moreover, they were confirmed to be Cd-tolerance genes *in planta* through lesion analysis in Cd-infiltrated leaves transiently expressing them. Taken together, our results demonstrate that maize *ASRs* play important roles in Cd tolerance, and they could be used as promising candidate genes for further functional studies toward improving the Cd tolerance in plants.

## 1. Introduction

Cadmium (Cd) not only negatively affects plant growth and development, but also for the human health hazards as the toxic elements usually accumulate in the consumable parts of crop plants [1,2,3,4]. Being a nonessential metal, Cd interferes with many cellular functions leading to retardation of plant growth, leaf chlorosis and a decrease in photosynthesis rate, resulting in the diminished water and nutrient uptake [5].

The mechanisms for plants to alleviate Cd stress vary from exclusion, compartmentation, and the synthesis of stress-related proteins [3,6]. Although some progresses have been made in exploring the genes associated with Cd detoxification and tolerance, the underlying molecular mechanisms of Cd tolerance in crops require further elucidation through analysis of novel candidate genes.

The plant-specific ABA-stress-ripening (ASR) gene family is well established for its response to ABA and multiple abiotic stresses, including drought, salinity as well as Al exposure [7,8,9]. ASR proteins are exclusive to the plant kingdom (albeit absent in *Arabidopsis*), and they play crucial roles in abiotic stress tolerance in most plants, as observed for *OsASR1*, *ZmASR1*, and *TaASR1* [10,11,12,13].

A plethora of studies on the heterologous and homologous expression of *ASR* genes in plant species were reported for functional characterization of the positive roles of plant ASRs in adaption to abiotic stresses. Overexpression of rice ASRs (*OsASR1* and *OsASR3*) and *OsASR2* in rice enhanced drought/salt tolerance and disease resistance/drought tolerance, respectively [14,15]. Heterologous expression of wheat *TaASR1*, foxtail millet (*Setaria italica*) *SiASR1* and *SiASR4*, confer drought tolerance in transgenic plants through regulating the antioxidant system [16,17,18], as was the case for overexpression of *Brachypodium distachyon BdASR1* in tobacco [19]. Akin to the performance of aforementioned cereal ASRs, overexpression of banana ASR (*MaASR*) in *Arabidopsis*, *Salicornia brachiata SbASR1* in groundnut, and soybean *ASR* in hairy roots enhanced drought and/or salt tolerance [20,21,22]. Tomato plants overexpressing *ASR1* displayed tolerance to water stress, whereas *ASR1*-antisense plants sensitive to water stress [23]. Plantain (*Musa paradisiaca*) MpASR protein may act as an osmoprotectant and water-retaining molecule to help cell adjustment to osmotic stress in transgenic *Arabidopsis* [24]. Similarly, lily ASR may act as an osmoprotectant as well as a transcription factor to confer the enhanced resistance against cold and freezing in transgenic *Arabidopsis* plants [25].

ASRs are dual target proteins that participate as chaperones in the cytoplasm and as transcription factors in the nucleus [11]. In maize, ZmASR1 acts both as a transcriptional regulator and as a chaperone-like protein [13]. Rice transcription factor *ASR5*, *the ZmASR1* ortholog, plays multiple roles in response to drought stress by regulating ABA biosynthesis, promoting stomatal closure, as well as acting as chaperone-like protein that possibly prevents drought stress-related proteins from inactivation [7].

It has been well established that some ASR proteins are known to be modulated by binding to metal ions [16]. Most ASR proteins have been shown to possess a zinc-binding domain [17,26]. Tomato ASR1 affects plant metabolism by its dual activity as a chaperone-like protein in the cytosol and as a transcription factor in the nucleus after acquiring its quaternary structure upon addition of Zn^2+^ [27,28,29], and similar results of Zn-induced structural transition were observed for HvASR1 and TtASR1 [30]. Intriguingly, soybean GmASR protects against oxidation damage by buffering metal ions, thus alleviating metal toxicity in plant cells under stressed conditions [16]. Rice ASR1 and ASR5, co-localized in nuclei and cytoplasm, are complementary transcription factors regulating Al-responsive genes to provide Al tolerance in rice [11,23,24,31].

Although these studies have unraveled the roles of ASRs in abiotic stress tolerance in plants, very limited information is available on ASRs that regulate the heavy metal detoxification and tolerance. Most ASR proteins have been shown to possess a zinc-binding domain [17,26], and plant ASRs can buffer metal ions [16,32]. Cd exhibits high chemical similarity with Zn and its uptake occurs through transporters engaged in the uptake of Zn [33]. Therefore, it is of particular interest to characterize ASRs in Cd tolerance. In the current study, based on the preliminary identification of Cd-tolerance related *ZmASR1* through survival screening of yeast cDNA expression library clones, four maize *ASR* members were cloned and testified for Cd tolerance using yeast complementation assay. Then they are transiently expressed in tobacco leaves for subcellular localization assay and Cd challenged tobacco lesion analysis, continuously. Comprehensive elucidation of these *ASR* genes associated with Cd tolerance in maize will pave the way for future studies aimed at unveiling the molecular mechanisms involved in regulating Cd tolerance.

## 2. Results

### 2.1. Candidate Cd Tolerance Gene ZmASR1 *Isolated from the Maize* cDNA Library

Yeast-based functional gene hunting is a powerful tool for identifying genes associated with stress tolerance [34]. Particularly, Cd tolerant genes can be identified through rescue assay by expressing cDNA library in Cd-sensitive yeast mutant *Δycf1* [35,36,37].

To identify novel plant genes that confer Cd tolerance, a maize leaf cDNA library used for yeast two-hybrid assay was utilized directly for screening. Approximately 10^4^ yeast colonies were screened, and a series of colonies survived in the presence of 100 μM CdCl_2_ and the respective inserts in the library vector were sequenced. Among the putative Cd-tolerance genes output by this shotgun approach, a transcript of particular interest displayed a high homology to *GRMZM2G136910*, which is annotated to be stress-responsive *ZmASR1* (Abscisic Acid, Stress and Ripening protein). Further sequence analysis showed that this Cd-tolerant clone contained the complete CDS of *ZmASR1* (Supplementary File 1), implying that this cDNA library is suitable to screen the Cd-tolerant *ZmASR1* with full-length CDS.

### 2.2. Sequence Analysis of Maize ZmASR Genes

**Figure 1:**
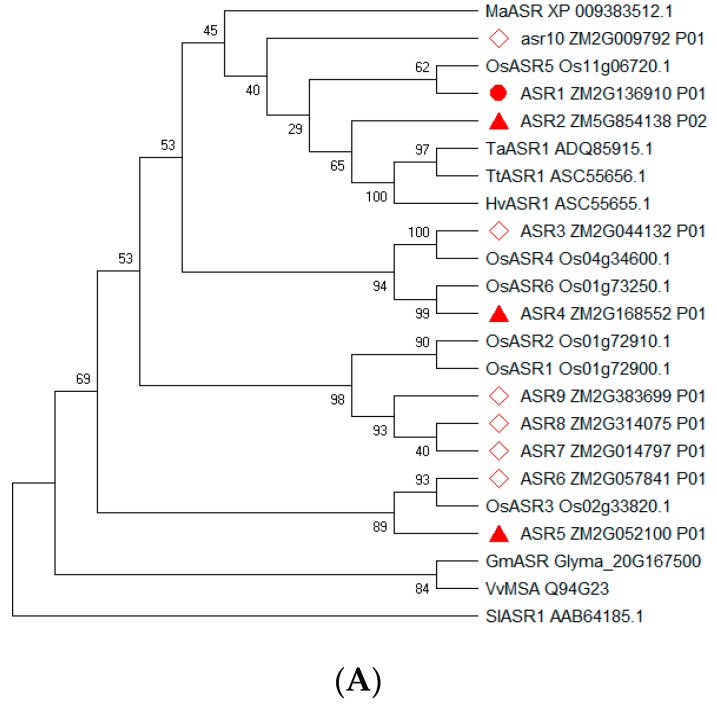

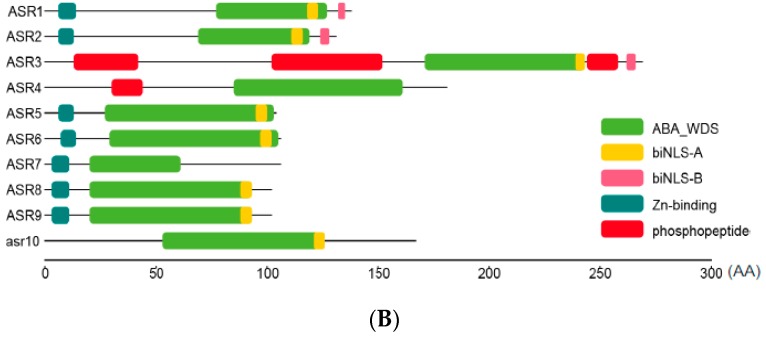
Phylogenetic relationships and conserved domains of maize ASRs. (**A**) Phylogenetic relationships of the maize ASRs and their homologs in grass species. Multiple alignments of ASR protein sequences were carried out with ClustalW utility embedded in MEGA7 software [40]. Phylogenetic trees were constructed by MEGA7 software using the neighbor-joining (NJ) method with 1000 bootstrap replicates, and tomato SlASR1 was used as the outgroup. Maize ASR1 was marked with one red dot, while other three maize ASRs for further experimental investigation were prefixed with triangles, and the left maize ASRs were marked with red diamonds. The ASRs in other plant species are prefixed as follows: Gm for soybean, *Glycine max*; Hv for barley, *Hordium vulgare*; Ma for banana, *Musa acuminate*; Os for rice, *Oryza sativa*; Sl for Tomato, *Solanum lycopersicum*; Ta for common wheat, *Triticum aestivum*; Tt for durum wheat, *Triticum turgidum*; Vv for grape, *Vitis vinifera*. (**B**) Domain organization of ZmASRs. The zinc-binding region, ABA/WDS domain, and the putative nuclear targeting signal were indicated in different color.

*Nine ZmASR* genes were identified in the maize genome previously, based on the presence of abscisic acid (ABA)/water deficit stress (WDS) signature [13], and they were named *aasr1* through *aasr9* in the latest version of maizeGDB [38] (https://maizegdb.org). In addition, GRMZM2G009792 encoding ABA/WDS domain protein with peptide evidence [38,39] (https://maizegdb.org) was designated as *asr10* in the current study. Multiple amino acid sequence alignment and phylogenetic relationships of the maize ASRs and their homologs indicated that ASR1, ASR3, ASR4, and ASR2, were related to rice OsASR5, OsASR4, OsASR6, and barley HvASR1, respectively, while ASR5 and ASR6 showed high homologous to rice OsASR3 (Figure 1A).

ZmASR1 to ZmASR3 protein contained conserved ABA/WDS domains and bipartite nuclear localization signals. With the exception of ZmASR3, ZmASR4 and asr10, all of the maize ASRs contained a small N-terminal consensus containing a stretch of six His residues which was typical for Zn-binding [12,17,26] (Figure 1B).

### 2.3. Expression Patterns of ZmASR Genes

**Figure 2:**
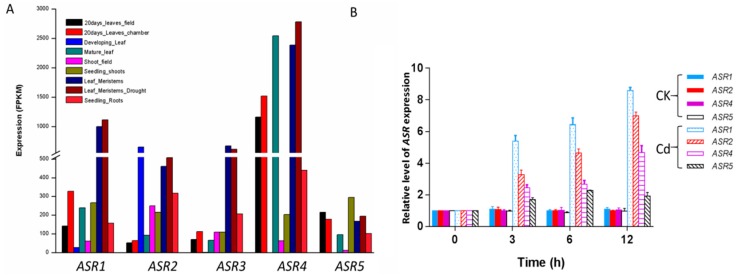
Expression patterns of maize *ZmASR* genes. (**A**) In silico analysis of the expression of maize *ZmASR* genes in different tissues. Maize transcript profiles were retrieved from five transcriptomic studies (Supplementary Table S1) hosted in qTeller (http://qteller.com). Gene expression values were represented by FPKM. (**B**) Expression analysis of *ASRs* in leaves of maize plants exposed to Cd treatment. Maize plants were treated with Cd(NO_3_)_2_ for various times as indicated. The relative expression levels of genes was analyzed by real-time qRT-PCR. Values are means ± SE of three independent experiments.

To investigate the potential functions of the *ZmASR* genes, the expression profiles of the *ZmASR* family members were generated using RNAseq data of three tissues (root, stem, and leaf) from maize gene expression omnibus (http://qteller.com) (Figure 2A, Supplementary Table S1). Basically, *ZmASR* genes can be categorized into two groups according to their expression patterns in different tissues.

The highest expression of *ZmASR6* to *ZmASR9* was less than 50 FPKM (fragments per kilobase transcript per million reads mapped), and *asr10* < 100 FPKM, whereas those of *ZmASR1* to *ZmASR4* were more than 500 FPKM in the investigated tissues (Figure 2A, Supplementary Table S1). Consistent with this, proteomic analysis through 2-DE condition allowed the identification of these 5 maize ASRs [13]. However, a close examination of the genomic structure of *ZmASR3* revealed the presence of cis-NAT (cis-natural antisense transcript) *GRMZM5G806182* transcribed from the opposite strand [38] (https://maizegdb.org), implying the complexity of *ZmASR3* locus. Therefore, four *ASR* members (*ZmASR1* to *ZmASR5* except *ZmASR3*) were selected for further analysis.

To gain insight into the functional significance of these 4 *ASRs* in Cd stress acclimation, we further explored the expression pattern of them after Cd treatment in maize leaves using qRT-PCR. A significant increase in the expression of *ASRs* was observed after 3 h of Cd treatment, and their expression was kept at a relatively high level in comparison with the untreated plants during the 12 h of Cd stress (Figure 2B).

### 2.4. Confirmation That Maize ASRs Confer Cd Tolerance in Cd-Sensitive Yeast Mutant

To avoid the Cd-tolerance achieved from co-transformation with multiple different genes in yeast during the library screening mentioned above, we reconstructed the CDS sequence of *ZmASR1* into the yeast expression vector pYES2 and retransformed to Cd-sensitive yeast *Δycf1* cells to test whether *ZmASR1* can complement *Δycf1* phenotype, thus confirming its functionality of Cd-tolerance identified by gene-hunting method. To investigate whether other *ASRs* hold the similar function, *ZmASR2* to *ZmASR5* except the neglected *ZmASR3* were cloned and evaluated their functionality of Cd-tolerance in yeast.

**Figure 3:**
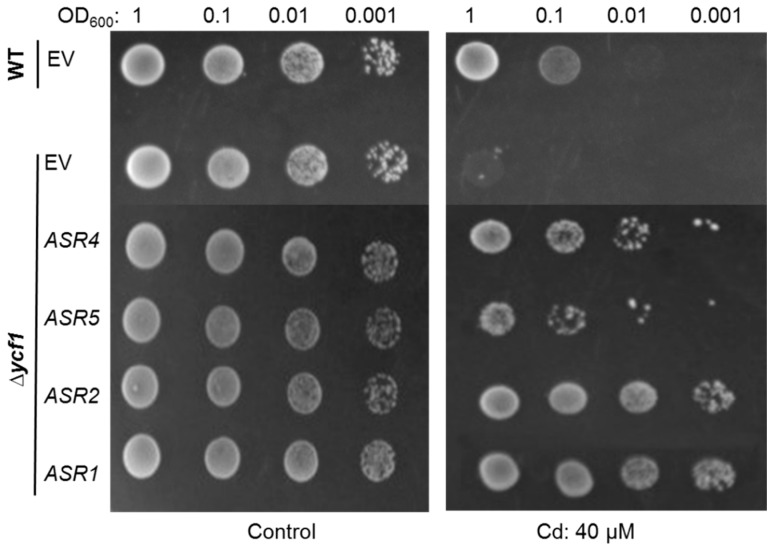
Maize *ZmASR* genes confer Cd-tolerance in yeast. The survival test of yeast strains transformed with maize *ZmASR* or pYES2 empty vector (*EV*) on SG-Ura agar medium supplemented with 40 μM CdCl_2_ in the presence of 2% galactose. Yeast cells transformed with *EV* were used as control.

The results showed that both *Δycf1* strains expressing the *ZmASRs* and the pYES2 empty vector (*EV*) grew well under non-stressed conditions. However, the dilution spot tests showed that *Δycf1* cells expressing *ZmASRs* exhibited dramatically enhanced growth when they were compared with *Δycf1* cells transformed with *EV* on half-strength SG agar medium supplemented with 100 μM CdCl_2_. The growth of *Δycf1* cells expressing *ZmASRs* were even better than that of wild-type yeast cells transformed with *EV* under Cd stress. Moreover, the *Δycf1* mutant transformed with *ZmASRs* constructs were able to survive at different dilutions whereas the growth of control was arrested (Figure 3). These data indicated that *ZmASR1* to *ZmASR5* except *ZmASR3* confer Cd-tolerance in yeast, though *ASR5* exhibited weak Cd-tolerance in yeast.

### 2.5. Transient Expression of Maize ASRs Conferred Cd Tolerance in Tobacco

**Figure 4:**
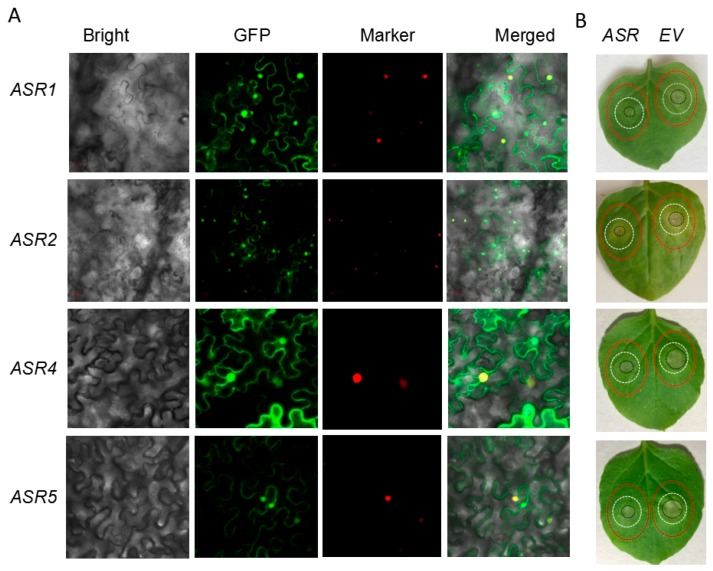
Subcellular localization and Cd-tolerance assay of GFP-tagged ZmASR fusion proteins in *N. benthamiana*. (**A**) Subcellular localization of GFP and the ZmASR-GFP fusion protein in *N. benthamiana* leaf cells. Epidermal cells of *N. benthamiana* leaves transiently expressing GFP fusion proteins were observed using confocal microscopy at 4 days post Cd infiltration. RFP-H2A, localized in the nucleus, was used to indicate the nuclei [41]. (**B**) Lesions were photographed at 4 days post Cd treatment. The *EV* transformed control region is on the right, while region transient expressing *ZmASR* is on the left for each leaf. Agro-infiltrated areas are indicated by red circles and Cd-infiltrated areas are circled by the smaller white lines, whereas Cd-caused lesions are indicated by the internal black circles on each leaf.

ZmASR1 to ZmASR3 harbored bipartite nuclear localization signal [13], and they were predicted to be localized to nucleus by Protcomp-PL (http://www.softberry.com/cgi-bin/programs/proloc/protcomppl.pl). To further study the functions of the maize *ASR* genes in response to Cd stress *in planta*, we currently focused on the characterization of *ZmASR1 to ZmASR5 except ZmASR3*, and a transient expression assay was performed with the ZmASR-GFP fusion protein in tobacco leaves. The results indicated that these four ASR-GFP fusion proteins were broadly distributed in the nucleus and cytoplasm (Figure 4A).

To further assess the functional relevance of these 4 maize *ASRs in planta* against Cd stress, tobacco leaves transiently expressing them were treated by infiltrating Cd solution, and the leaf regions transiently expressing empty vector (*EV*) were used as control. The *EV* transformed control regions showed obvious chlorosis and lesions 4 d after Cd treatment, indicative of a symptom of Cd toxicity. However, lesions on leaves transiently expressing *ZmASR* were significantly smaller than those on leaves expressing *EV* post Cd inoculation (Figure 4B). Therefore, these results suggested that transient expression of maize *ASR* also conferred Cd tolerance in tobacco cells.

## 3. Discussion

### 3.1. Maize ASRs Function in Cd-Tolerance

Some poaceae ASR members, including maize *ASR1* and rice *OsASR5*, are the most prevalent ASR proteins in major plant tissues, and large-scale quantitative proteomics studies only revealed ASR1 to ASR5 in maize leaves [42,43]. Interestingly, maize ASR1, encoded by one of the most highly expressed *ZmASR* genes, was the most abundant detected ASR protein in maize leaves [13]. In the current research, among the 10 *ASR* isoforms, only *ASR1* was easily identified to be Cd tolerant through survival screening of the maize leaf cDNA library hosted in yeast (Supplementary File 1). Moreover, the ectopic expression of maize *ASRs* including *ASR1* enhanced Cd tolerance in both tobacco leaves and yeast (Figure 3 and Figure 4). Several studies on the heterologous and homologous expression of *ASR* genes in plant species have suggested that *ASRs* increase abiotic stress tolerance, as observed for *OsASR1*, *ZmASR1*, *TaASR1* and *SiASR4* [1,12,13,17]. These together strengthened the positive roles of plant ASRs in adaption to abiotic stresses.

However, the study on *ASRs* that respond to ions or regulate the heavy metal detoxification and tolerance is in its infancy. It was speculated that the expression of *GmASR* might be upregulated to buffer the concentration of Zn^2+^, thus alleviating metal toxicity in plant cells under stressed conditions [16]. These implicated that the functions of some ASR proteins are known to be modulated by binding to metal ions [16].

In the current study, *ZmASR1* was identified to be putative Cd-tolerant by yeast based-gene hunting approach. Then the maize paralogs of *ASR1* were cloned and subjected to Cd-tolerance investigation, and the results showed that the ectopic expression of maize *ZmASR2* to *ZmASR5* except *ZmASR3* enhanced Cd tolerance in yeast (Figure 3). Moreover, the selected 4 maize *ASR* members of *ASR1* through *ASR5* conferred Cd-tolerance in tobacco via transient expression assay (Figure 4B).

This is reminiscent of soybean GmASR alleviating metal toxicity [16] and rice ASR1 (Os02g33820) together with ASR5 (Os11g06720) regulating Al responsive genes [11]. GmASR protects against oxidation damage by buffering metal ions, thus alleviating metal toxicity in plant cells under stressed conditions [16], while rice transcription factors *ASR1* and *ASR5* act in concert and complementarily to regulate Al responsive genes, and participate as chaperones in the cytoplasm by virtue of dual target proteins [11]. Notably, the Cd-tolerant ZmASR1 and ZmASR5 were closely related to the coupled rice *ASR5* and *ASR1* involving in Al-tolerance, respectively (Figure 1A), raising the interesting possibility that ZmASR1 and ZmASR5 act synergistically to facilitate the Cd-tolerance. A better understanding of the coordinated roles of these maize *ASR* genes in stress acclimation will require more exhaustive exploration of their targets and their chaperone-like activities using the genetic engineering plants subjected to metal pressure.

Interestingly, rice ASR (Os11g06720, the above-mentioned ASR5) with reactive oxygen species (ROS) scavenging and chaperone-like activities enhances acquired tolerance to abiotic stresses through induction of various cell rescue proteins in yeast cells [44]. In the present study, the Cd-tolerant Zm*ASR1* identified via yeast rescue screening displayed high identity to the aforementioned dual-function rice ASR5 (Os11g06720) (Figure 1A). Analogously, a very likely explanation is that Zm*ASR1* acts as a transcription regulator modulating gene expression and a protective molecule under Cd stress. Further assays by means of physiological characterizing its transgenic plants might help to elucidate this point.

### 3.2. The Feature of Dual Targeting Determines the Multiple Function of ASRs

Most ASR proteins have been shown to possess a zinc-binding domain at the N-terminal end and a putative nuclear targeting signal at the C-terminal end [17,26]. However, ASRs display different subcellular localizations.

Some ASRs are dual target proteins that participate as chaperones in the cytoplasm and as transcription factors in the nucleus [11]. While some ASR proteins are found exclusively in the nucleus, where they act as transcription factors regulating gene expression during stress response, other ASR proteins are localized in both the nucleus and the cytosol, likely reflecting their diverse functions [17,19,30].

Zinc is an essential cofactor and is bound by proteins through coordination by the amino acids His, Asp, Glu and Cys. Intriguingly, these zinc coordinating residues except Cys appear in high copies in ASRs [45]. Furthermore, the DNA binding of tomato SlASR1 is sequence specific and Zinc-dependent [32]. Wheat TaASR1 contains a N-terminal stretch of six His residues which is responsible for its transcriptional regulation activity, implying that TaASR1 may act as a transcription factor and bind to DNA in a Zn^2+^-dependent manner [12]. Another typical stretch (PEHAHKHK) with Zn^2+^-binding capacity was identified in barley HvASR1, durum wheat TtASR1 [30] and tomato SlASR1 [45]. It is noteworthy that these two motifs are also conserved in maize ASR1 and ASR2 (Figure 1B).

It was speculated that plant ASRs could buffer high Zn^2+^ concentrations that may accumulate in the cytoplasm under abiotic stress, giving time for cation-efflux transporters to restore the Zn^2+^ balance [32]. Cd exhibits high chemical similarity with functionally active ions situated in active sites of enzymes and signaling components, in particular Zn. Since Cd uptake occurs through transmembrane carriers engaged in the uptake of essential metallic elements (e.g., Ca, Fe, Mg, and Zn), it often induces mineral deficiencies by competing with the uptake of aforementioned essential elements [33]. From this perspective, it is of particular interesting to investigate whether Cd affects the activity of maize ASRs, the potential transcriptional regulator, and how ASRs regulate the divalent transporters responsible for the uptake of essential mineral elements.

Tomato ASR1, an intrinsically disordered protein (IDP), affects plant metabolism by its dual activity as a chaperone-like protein in the cytosol and as a transcription factor in the nucleus after acquiring its quaternary structure upon addition of Zn^2+^ [27,28,29,32]. Similar results of Zn-induced structural transition were observed for HvASR1 and TtASR1 [30]. In the current study, subcellular analysis indicated that ASR1, ASR2, ASR4 and ASR5 were distributed in cytoplasm and nucleus, which was consistent with the results in aforementioned literature.

The folding behavior determines the function of tomato ASR1 as one chaperone and/or transcription factor, and the binding of Zn^2+^ promotes its partial folding as an α-helix monomer, a prerequisite to bind its specific DNA sequence [32]. Tomato ASR1 may act as a transcription factor when adopting the adequate α-helix conformation, and its chaperone role is not affected by ASR1 structuring upon addition of Zn^2+^ [32]. Furthermore, Cd can replace Zn in proteins [2], and cadmium ions can induce the folding and dimerization of a designed metalloprotein [46]. Regarding the 4 dual-targeting maize ASRs, here we hypothesized that they function as transcription factors after the Zn analog Cd-binding promoted α-helix conformation, and as chaperones which behavior persist under Cd stress at micromolar concentrations of Cd ions. Further studies will be required to reveal the functional impact of the structural transitions that these proteins undergo in the presence of Cd, using the structural biological techniques reported previously [27,30,32], thus provide further insight into the precise character of ASR-mediated Cd-tolerance.

## 4. Materials and Methods

### 4.1. Isolation of Cd-Tolerance Genes from the Maize Leaf cDNA Library

For the identification of Cd tolerance genes, a maize leaf cDNA library constructed into pDEST22 hosted in *Saccharomyces cerevisiae* strain MaV203 for yeast two-hybrid assay (Invitrogen) [47] was utilized directly for screening. Colonies that grew on medium containing 100 μM CdCl_2_ were selected, and the pDEST22 plasmids carrying putative Cd-tolerance genes were isolated from these yeast cells and transformed to TOP10 *Escherichia coli* cells for sequencing the inserts (Invitrogen). The function annotation of the inserts sequence was performed following the description of the homologous genes output by the BlastX utility.

Then Cd tolerance function of the isolated plasmids was confirmed by repeating the transformation of the *Δycf1* cells [37,48].

### 4.2. Maize Seedlings Cultivation and cDNA Synthesis for Cloning ASR

The seedlings of maize (*Zea mays* L. inbred line B73) were cultivated in a hydroponic system in a growth chamber and continuously aerated and renewed every 3 days. When the third leaves were fully expanded, the seedlings were transferred into fresh growing solutions containing 100 µM Cd(NO_3_)_2_. After 3, 6 and 12 h of Cd treatment, maize seedlings leaves were sampled for RNA isolation.

Total RNA was extracted from leaves using the RNAiso Plus (TaKaRa Bio Inc., Dalian, China) according to the manufacturer’s instructions. Approximately 2 µg of total RNA was reverse transcribed using oligo d(T)_16_ primer and M-MLV reverse transcriptase (TaKaRa). The synthesized cDNA was used for amplifying the coding sequences (CDS) of *ASR*.

The expression levels of maize *ASRs* were measured by qRT-PCR using a DNA Engine Opticon 2 real-time PCR detection system (Bio-Rad) with SYBR Premix Ex Taq (TaKaRa). The expression level of each target gene was normalized against that of *ZmActin* in maize.

### 4.3. Sequence Alignment and Phylogenetic Analysis

Multiple alignments of ASR protein sequences were carried out with ClustalW utility embedded in MEGA7 software (Tokyo Metropolitan University, Tokyo, Japan) [40]. Phylogenetic trees were constructed by the neighbor-joining (NJ) method using MEGA7 software and the bootstrap tests were performed with 1000 replications.

### 4.4. In Silico Expression Analysis

We perform in silico experiments using maize transcript profiles from qTeller (http://qteller.com), which hosted about 40 experiments from nine sources, and the data of three tissues (leaf, root and shoot) from five transcriptomic studies (Supplementary Table S1) were retrieved for analysis.

### 4.5. Yeast and Transient Plant Expression Vector Reconstruction

To avoid the Cd-tolerance achieved from co-transformation with multiple different genes in yeast, we subcloned the coding sequences of *ZmASR1*, together with other 4 maize *ASRs*, into the yeast expression vector pYES2 and binary vector pSuper1300-GFP with the primers listed in Table 1.

### 4.6. Cd Tolerance Complementation Assay in Cd-Sensitive Yeast

The *ZmASRs* recombinant plasmids and pYES2 empty vector (*EV*) were then transformed into Cd-sensitive yeast *Δycf1* mutant cells using the lithium acetate/PEG transformation method, and the isogenic yeast wild-type BY4741 (*MATa*; *ura3Δ0*; *leu2Δ0*; *his3Δ1*; *met15Δ0*) [49] transformed with *EV* as positive control [50,51,52,53,54,55].

Yeast strains expressing *EV* or *ZmASRs* were pre-cultured in SD-Ura liquid medium to an optical density at 600 nm (OD_600_) of 1.0, and 10-μL of tenfold serial dilutions were spotted onto SD-Ura agar medium with or without 40 μM CdCl_2_ in the presence of 2% galactose. After incubation at 30 °C for 3 days, the growth of clones transformed with putative Cd-tolerance maize genes was compared with those transformed with the *EV* on the same plates supplied with Cd [37,52]. All drop-test experiments were independently repeated at least three times.

### 4.7. Agro-Infiltration and Cd Response in Tobacco Leaves

Agro-infiltration and Cd treatment assay was performed based on previous reports [4,54,55,56,57,58], with minor modifications. *Agrobacterium tumefaciens* strain GV3101 was transformed with *ZmASRs* constructs and then grown in Luria-Bertani culture medium supplemented with appropriate antibiotics. After 36–48 h, *A. tumefaciens* cells were spun down by centrifugation, and re-suspended in Agro-infiltration buffer (10 mM MgCl_2_ and 10 mM 2-(N-morpholino) ethanesulfonic acid (MES), pH 5.6). The re-suspended *A. tumefaciens* cells were diluted and mixed with P19 silencing suppressor in a 1:1 to a final OD_600_ = 0.3 for each construct before infiltrating into the leaves of 3–4 week-old tobacco (*Nicotiana benthamiana*) plants.

After 3 days of agro-infiltration, tobacco leaves transiently expressing ZmASRs*-GFP fusion proteins* were analyzed using confocal fluorescence microscopy to monitor transformation. For fluorescence observations, patches were cut from tobacco leaves 3 days after agro-infiltration and used for confocal imaging on a Zeiss LSM 710 confocal laser scanning microscope. RFP-H2A, localized in the nucleus, was used to mark the nuclei [41]. GFP fluorescence was excited by the 488 nm line of an argon laser, and emissions were detected between 500 and 530 nm. Simultaneously, the needle hole in the leaves expressing ZmASRs*-GFP* were re-infiltrated with 500 μM Cd(NO_3_)_2_. The infected leaves were photographed were measured at 4 days post-treatment.

Each experiment was repeated at least three times with a minimum of 10 infected leaves. Leaf regions transiently expressing EV were used as a control.

## Figures and Tables

**Table 1:** Primers for construction of *ZmASRs* in yeast and tobacco transient expression vectors.

**Gene**	Gene Model *	**pYES2-F (5′-3′)**	**pYES2-R (5′-3′)**
*ZmASR1*	ZM2G136910	AAGCTT-AATTGTCACTTGCTCTCC	GGATCC-GCTCGATATCACTCTCAC
*ZmASR2*	ZM5G854138	AAGCTT-CCAGCCATCCTACTGTCACA	GGTACC-TCAGCCGAAGAGGTGGTGGT
*ZmASR4*	ZM2G168552	AAGCTT-TGAGAGCCTGAGACGATGG	GGTACC-TTCAGTCGCAGTAGTAGGAGTA
*ZmASR5*	ZM2G052100	AAGCTT-GGAGCCATGTCTGAGGAGAAG	GGTACC-CGACGATGTGCTGCTGCTT
		**pSuper-1300-F (5′-3′)**	**pSuper-1300-R (5′-3′)**
*ZmASR1*	ZM2G136910	TCTAGA-ATGGCGGAGGAGAAG	GGTACC-GCCGAAGAAGTGGTG
*ZmASR2*	ZM5G854138	AAGCTT-CCAGCCATCCTACTGTCACA	GGTACC-GCCGAAGAGGTGGTGGTG
*ZmASR4*	ZM2G168552	AAGCTT-TGAGAGCCTGAGACGATGG	GGTACC-GTCGCAGTAGTAGGAGT
*ZmASR5*	ZM2G052100	AAGCTT-GGAGCCATGTCTGAGGAGAAG	GGTACC-GTTGTGGGCGTGCTTCTT

* Gene model is shown in abbreviated form through slicing the prefix GRM.

## Supplementary Materials

The following are available online at http://www.mdpi.com/1422-0067/20/1/133/s1, Supplementary File 1 The sequence information of the Cd-tolerance clone isolated from the maize cDNA library. Supplementary Table S1. Expression profiles of maize *ZmASR* genes in different tissues.
