# Supplementary material for: Comprehensive Analysis of the Cadmium Tolerance of Abscisic Acid-, Stress- and Ripening-Induced Proteins (ASRs) in Maize
Source: Int J Mol Sci. 2019 Jan 1;20(1):133. doi: 10.3390/ijms20010133 (PMC6337223; doi:10.3390/ijms20010133)
Supplement: Supplementary file 1 [file ijms-20-00133-s001.pdf]

Supplementary File1 The sequence information of the Cd-tolerance clone isolated from the maize cDNA library.

>M9-22F_E11.ab1 "asr1"

TCTCGCACGTACAGCGNGATGATGTATATAACTATCTATTCGATGATGAAGATACCCCACCAAACCCAAAAAA

AGAGGGTGGGTCGAATCAAACAAGTTTGTACAAAAAAGTTGGCGATCCAATTGTCACTTGCTCTCCCTCCAACAAGCTAA

TTAAGGCCGGTCCATCCCTCTTCTAGCTCGTTTCATTATCCATGGCTGAGGAGAAGCACCACCACCACCACCTGTTCCAC

CACAAGAAGGACGAGGAGCAGGCCGCGGCCGGCGGGTACGGCGAGTCCGCCGAGTACACGGAGGCCACGGTGACGGAGGT

CGTGTCCACGGGCGAGAACGAGTACGACGAGTACAAGAAGGAGGAGAAGCAGCACAAGCACAAGCAGCACCTCGGCGAGG

CCGGCGCCATCGCCGCCGGCGCCTTCGCACTCTACGAGAAGCACGAGGCAAAGAAGGACCCGGAGCACGCGCACCGCCAC

AAGATCGAGGAGGAGGTCGCGGCGGCGGCGGCCGTCGGCTCCGGCGGCTTCGCCTTCCACGAGCACCACGAGAAGAAGAA

GGACCACAAGGACGCCGAGGAGGCCGGCGGCGAGAAGAAGCACCACTTCTTCGGCTGATTGATCCCTCCCGTATCGTCGT

CCCTCCCCGTGTGCTACGCGTGCGTGTGTGAGAGTGATATCGAGCGCCCGCCGTGTTGTGCGCGCGTACGTATGTATGCG

CTCGTGTGATGCACGAATAAGCGTGGCTACGTAATCTATCGTATGTATACGTGTGTGTATGCATGTGCTTGTGTATGATC

GTGGTACGAGGACCGAAAAAATGTATGCAACTCTGATTTACTTAT

>M9-22F_E11.ab1 ASR1 (translated +3 frame)

SHVQRDDVYNYLFDDEDTPPNPKKEGGSNQTSLYKKVGDPIVTCSPSNKLIKAGPSLF*LVSLS

MAEEKHHHHHLFHHKKDEEQAAAGGYGESAEYTEATVTEVVSTGENEYDEYKKEEK

QHKHKQHLGEAGAIAAGAFALYEKHEAKKDPEHAHRHKIEEEVAAAAAVGSGGFAFHEHH

EKKKDHKDAEEAGGEKKHHFFG*

LIPPVSSSLPVCYACVCESDIERPPCCARVRMYALV*CTNKRGYVIYRMYTCVYACACV*SWYEDRKNVCNSDLLX

>ZmASR1/GRMZM2G136910_P01

MAEEKHHHHHLFHHKKDEEQLAAGGYGESAEYTEATVTEVVSTGENEYDEYKKEEK

EHKHKQHLGEAGAIAAGAFALYEKHEAKKDPEHAHRHKIEEEVAAAAAVGSGGFAFHEHH

EKKKDHKEAEEAGGEKKHHFFG

Supplementary Table S1. Expression profiles of maize ZmASR genes in different tissues.

Maize gene	Zm00001d023529	Zm00001d004843	Zm00001d003712	Zm00001d011813	Zm00001d025401	Zm00001d016760	Zm00001d040786	Zm00001d040785	Zm00001d040787	Zm00001d035409
Gene model	ZM2G136910	ZM5G854138	ZM2G044132	ZM2G168552	ZM2G052100	ZM2G057841	ZM2G014797	ZM2G314075	ZM2G383699	ZM2G009792
Gene name	*ZmASR1*	*ZmASR2*	*ZmASR3*	*ZmASR4*	*ZmASR5*	ZmASR6	ZmASR7	ZmASR8	ZmASR9	asr10
20days_leaves_field[a]	142.3	51.9	69.4	1162.7	214.9	1.4	1.6	0.0	0.7	56.3
20days_Leaves_gc(grow chamber)[a]	328.2	64.1	111.4	1519.6	177.7	2.7	5.2	0.2	0.7	64.1
Developing_Leaf[b]	26.6	648.7	0.4	0.3	0.0	8.2	0.7	0.4	2.4	7.6
Mature_Leaf[b]	239.9	92.0	65.7	2546.0	95.4	2.4	3.1	1.1	1.1	18.6
Shoot_field[c]	61.6	249.0	108.3	62.3	11.4	0.6	0.0	0.0	0.0	1.2
Seedling_shoots[d]	265.3	215.3	108.4	203.4	294.8	46.6	31.6	17.9	36.9	2.5
Leaf_Meristems[e]	1000.1	461.0	670.3	2385.2	166.7	9.8	2.9	3.7	5.1	91.0
Leaf_Meristems_Drought[e]	1115.7	508.1	615.3	2781.7	194.0	7.6	0.5	4.4	10.5	45.5
Seedling_Roots[d]	157.1	316.4	206.2	440.5	101.3	0.4	1.8	0.0	2.0	9.5

Maize transcript profiles were retrieved from five transcriptomic studies (marked with red lowercase letters) hosted in qTeller (http://qteller.com).

a, Davidson et al., 2011, Plant Genome, 4: 191-203. http://dx.doi.org/10.3835/plantgenome2011.05.0015.

b, Li et al., 2010, Nature Genetics volume 42, pages 1060–1067. http://dx.doi.org/10.1038/ng.703.

c, Chettoor et al., 2014, Genome Biology, 15:414; http://genomebiology.biomedcentral.com/articles/10.1186/s13059-014-0414-2.

d, Wang et al., 2009, Plant Cell, 21: 1053-1069; http://dx.doi.org/10.1105/tpc.109.065714.

e, Kakumanu et al., 2012, Plant Physiology, 160: 846-867; http://dx.doi.org/10.1104/pp.112.200444.

Gene expression values were represented by FPKM (fragments per kilobase transcript per million reads mapped).
